# Tracking mutation and drug-driven alterations of oncokinase conformations

**DOI:** 10.1007/s12254-021-00790-6

**Published:** 2022-01-21

**Authors:** Andreas Feichtner, Valentina Kugler, Selina Schwaighofer, Thomas Nuener, Jakob Fleischmann, Eduard Stefan

**Affiliations:** Institute of Biochemistry and Center for Molecular Biosciences, University of Innsbruck, Innrain 80/82, 6020 Innsbruck, Austria; Institute of Biochemistry and Center for Molecular Biosciences, University of Innsbruck, Innrain 80/82, 6020 Innsbruck, Austria; Tyrolean Cancer Research Institute, Innrain 66, 6020 Innsbruck, Austria

**Keywords:** BRAF inhibitor, MEK inhibitor, Cell based reporter assay, Cancer drug efficacy, Kinase biosensor, Personalized therapy

## Abstract

Numerous kinases act as central nodes of cellular signaling networks. As such, many of these enzymes function as molecular switches for coordinating spatiotemporal signal transmission. Typically, it is the compartmentalized phosphorylation of protein substrates which relays the transient input signal to determine decisive physiological cell responses. Genomic alterations affect kinase abundance and/or their activities which contribute to the malignant transformation, progression, and metastasis of human cancers. Thus, major drug discovery efforts have been made to identify lead molecules targeting clinically relevant oncokinases. The concept of personalized medicine aims to apply the therapeutic agent with the highest efficacy towards a patient-specific mutation. Here, we discuss the implementation of a cell-based reporter system which may foster the decision-making process to identify the most promising lead-molecules. We present a modular kinase conformation (KinCon) biosensor platform for live-cell analyses of kinase activity states. This biosensor facilitates the recording of kinase activity conformations of the wild-type and the respective mutated kinase upon lead molecule exposure. We reflect proof-of-principle studies demonstrating how this technology has been extended to profile drug properties of the full-length kinases BRAF and MEK1 in intact cells. Further, we pinpoint how this technology may open new avenues for systematic and patient-tailored drug discovery efforts. Overall, this precision-medicineoriented biosensor concept aims to determine kinase inhibitor specificity and anticipate their drug efficacies.

## Mutated kinase hubs

**Fig. 1:**
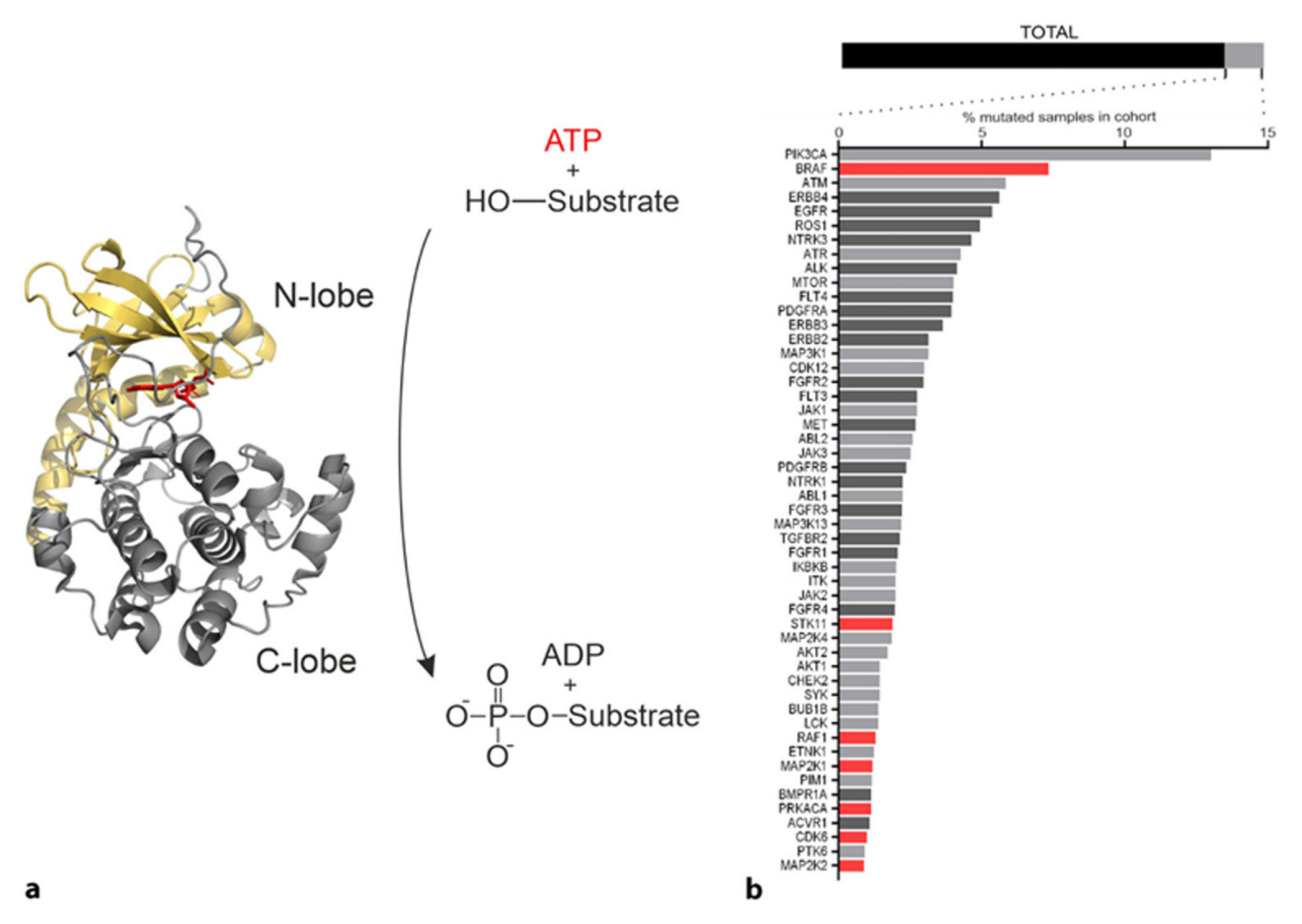
Kinase domain function and kinase mutations. **a** Exemplary a phosphotransferase reaction catalyzed by a kinase domain is illustrated. Conventionally, the respective kinase transfers the γ-phosphate of ATP to a hydroxyl group of either a serine, threonine or tyrosine residue of the substrate protein. We have illustrated the structure of the catalytic sub-unit of the cAMP-dependent protein kinase A (PRKACA, RCSB 4O21). The N-lobe is marked in *beige*, the C-lobe in *grey*, and the bound ATP in *red*. The image was created using PyMOL Molecular Graphics System, Version 2.1.1 Schrödinger, LLC, New York City, NY, USA. **b** Mutated genes (*576*) of a patient cohort consisting of 12,647 cases represented by the *bar* on top (TOTAL). Mutated kinases (52) are highlighted in *light grey*, the remaining mutated genes (*524*) are shown in *black*. Below the percentage of simple somatic mutations occurring for each kinase are shown. For the kinases highlighted in *red* Kin-Con biosensors are available. Kinases with transmembrane regions are highlighted in *dark grey*. Data were obtained at https://portal.gdc.cancer.gov/

Protein kinases represent one of the largest super families of human genes which encode over 500 functionally diverse enzymes [1, 2]. Kinases act as central units for signal propagation. Structurally, protein kinase domains comprise two lobes (the N-terminal lobe and C-terminal lobe) with the active site in a cleft between them. The alignment of hydrophobic key residues of the regulatory spine (R-spine) is a characteristic of the active kinase state, which is dynamically assembled as part of regulation. On the contrary, the catalytic spine (C-spine) is formed upon binding of the adenine ring of adenosine triphosphate (ATP) [3–5]. Conformational rearrangements of the kinase domain (the N:C lobes) are required for substrate binding, catalysis, and the subsequent product release. Dynamic intrinsic properties of the R/C spines are affected either through mutations or diverse types of molecular interactions with macromolecules, second messengers, or bioactive small molecules [6–9]. Conventionally, kinases catalyze the transfer of the γ-phosphate group from ATP to the hydroxyl group of serine, threonine, or tyrosine residues of substrate proteins (Fig. 1a). A highly dynamic and transient event, as counteracting activities of phosphatases revert this process and release the phosphate [10]. Thus, system-dependent input signals are sensed and lead to a switch-like signaling response by interconversion of active (ON-position) and inactive (OFF-position) conformation states [11].

Post-translational modifications (PTMs) and/or diverse types of (macro)molecular interactions form the basis for the ON–OFF dynamics of these molecular switches. While generally tightly regulated, mutations or gene amplifications of kinases lead to aberrant phosphorylation activities and thereby facilitate the onset of a plethora of human diseases, such as asthmatic, cardiovascular, or inflammatory diseases and cancer [2, 12].

Due to the prevalence of diseases associated with dysregulated kinase activities, protein kinases have emerged as central targets for pharmaceutical intervention ever since the US Food and Drug Administration (FDA) approval of imatinib as the first direct kinase inhibitor drug in 2001. As of early 2021, a total of more than 60 small molecule kinase inhibitors gained approval status, the major portion of which are prescribed for the treatment of neoplastic diseases [7]. Reflecting the success, but also draw backs, of kinase block-buster drug treatments, we would like to stress that the current understanding of kinase activation principles primarily involves the conserved kinase domain [13]. This is due to the fact that for many kinases full-length structures are still missing and compartmentalized molecular interactions are taken into account less often for kinase activity profiling. Physiological and pathological kinase functions, however, depend on intra- and intermolecular interactions of the full-length kinase entities. Kinase domain structures (crystals and the modelled ones) are sometimes not sufficient to predict the impact of mutations and lead molecule binding on signaling properties of the cellular kinase complex. Thus, context-dependent molecular kinase interactions remain poorly understood.

Specificities and efficacies are central aspects for the development of new kinase inhibitors. Poly-phar-macologically acting inhibitors like imatinib target a wide range of kinases [14]. These circumstances however endorse the risk of side effects. It was the identification of hotspot cancer driver mutations which opened a new avenue for kinase-directed therapy approaches. Vemurafenib, the first mutation-specific kinase inhibitor, was used for treatment of melanomas harboring a distinct genetic background [15]. This BRAF inhibitor (BRAFi) exhibits increased selectivity for the mutated oncokinase BRAF^V600E^ [16]. This point mutation occurs in 50% of malignant melanomas and represents the most frequent mutation of BRAF. More than 300 different BRAF mutations have been identified so far [17, 18]. Fig. 1b ranks kinases according to the frequency of reported mutations.

Exemplarily, we describe the physiological and pathological activation cycle of BRAF. Typically, RAF kinase activity is governed through the ligand-mediated activation of upstream receptor tyrosine kinases (RTKs) such as EGFR, which lead to the GTP-loading and thus activation of RAS and subsequent binding and activation of RAF. Activation of the three RAF isoforms ARAF, BRAF, and CRAF depend on the binary protein-protein interaction (PPI) with the GTP-activated, membrane bound RAS GTPases [19]. Subsequently, in a series of dimerization and phosphorylation events, the membrane-recruited RAF complex is released from the closed conformation and adopts the active, opened configuration [20–23]. Signal propagation to the downstream kinases MEK1/2 and ERK1/2 ultimately results in reorganization of nuclear gene expression programs to increase cellular proliferation or to influence differentiation. Hyperactivation of this pathway, primarily through gain-of-function mutations in *KRAS* or *BRAF*, are commonly described in melanoma, thyroid and colorectal cancers [13, 15]. Paradoxically, in certain genetic backgrounds, i.e., mutated RAS, application of BRAFi promotes pathway reactivation through different means, and thus boosts tumor progression [15, 24]. This underlines the necessity of personalized therapy approaches for defining individual concepts for target-oriented pharmaceutical interference.

## Personalized medicine

While next-generation sequencing technology already allows rapid genomic characterization of patient tumor samples and enables identification of cancer driver mutations, methods for fast prediction of drug efficacies for each unique genetic background are still lacking. The majority of currently applied technologies for determination of kinase activities rely either on purified kinases, commonly limited to the kinase domain, or cell lysates [25–29]. Disruption of the cellular environment, however, might have unforeseeable effects on kinase functions, as the concerted interplay between activating and deactivating inputs, provided by the cellular environment, represents the fundamental mode of kinase activity regulation. Therefore, live-cell based assays provide a more suitable approach while retaining the entirety of inputs necessary to better mimic the in vivo setting [25–30].

In this context it is of interest that many kinases share a similar concept for kinase regulation. This involves so-called intramolecularly acting cis-regulatory elements (CREs) or auto-inhibitory modules (AIM) which control substrate access and kinase activity states [30–33]. The kinase OFF state is characterized by interaction and association of the CRE with the catalytic cleft of the kinase domain. Thereby, this kinase conformation effectively blocks substrate binding and the subsequent phosphate-transfer reaction. Intramolecular reorganizations and thus disengagement of CRE and kinase domain, mediated through the aforementioned means, switches the kinase into the transient ON state.

Kinase patient mutations interfere with kinase activation cycles and lead in many cases to constitutive activation of kinase signaling. In contrast to kinase inactivating mutations a growing list of oncogenic kinase-activating mutations lead or contribute to carcinogenesis in different cancer settings [2, 34–36].

## KinCon: the kinase conformation reporter

**Fig. 2:**
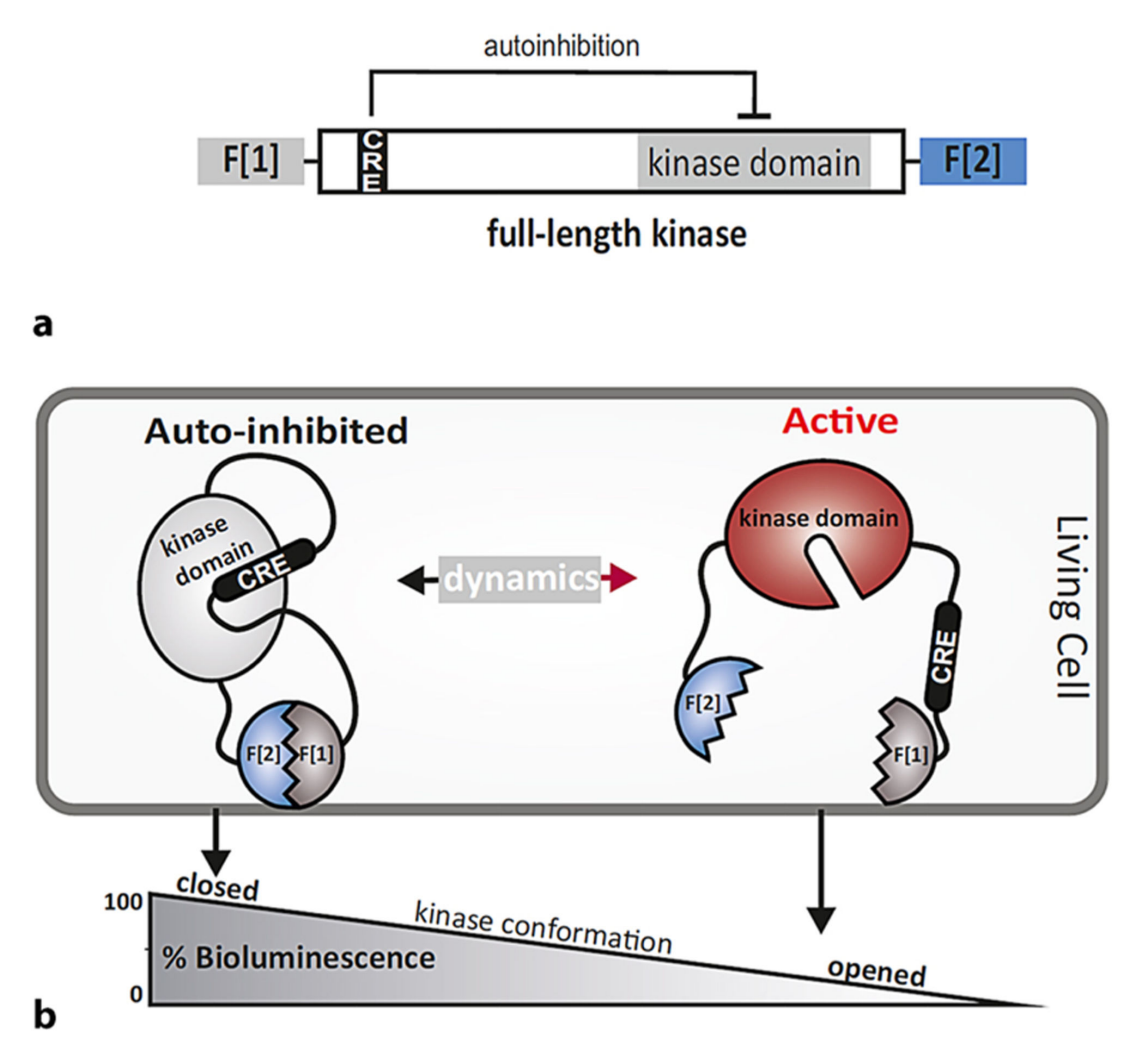
The KinCon biosensor concept. **a** Modular structure of the KinCon biosensor. Mammalian expression vectors encode for the full-length kinase sequence and flanked fragments of the luciferase PCA. Exemplarily, we show that the chosen full-length kinase contains one cis regulatory element (*CRE*). A flexible linker separates fragment 1 (–F[1]) and fragment 2 (–F[2]) of the luciferase PCA. **b** The opened and active full-length kinase conformation is adopted when –F[1] and –F[2] of the PCA-luciferase are spatially separated. In the presence of the respective luciferase substrate less or no bioluminescence is emitted. Conventionally, in a more closed kinase conformation, the kinase is less active or inactive. Thus, the two fragments are in close proximity to form a complemented and functional luciferase which catalyzes substrate conversion and consequently recordable light emissions. The different means leading to KinCon dynamics are discussed in relation to Fig. 3

We recently developed a genetically encoded kinase conformation (KinCon) sensor system, which can be used to track kinase activity states in living cells [22, 30, 33, 37, 38]. The KinCon biosensor has a modular structure and is generated by inserting the coding sequence of the full-length kinase of interest into a standardized eukaryotic expression vector. The insertion site is flanked by two linker sequences and two frag-ments (–F[1] and –F[2]) of a protein-fragment complementation assay (PCA) which is conventionally based on *Luciferase* enzymes ([39, 40]; Fig. 2a). The presented modular KinCon concept allows the generation of novel biosensors by replacing the respective coding region between the linker and the PCA fragments with the kinase of choice. The general concept of KinCon reporters is illustrated in Fig. 2b. Different means of upstream regulation along with oncogenic mutations may lead to kinase activation which is mostly reflected by conformational rearrangements of the full-length enzyme. These structural and enzymatic transformations need to be reversible under physiological conditions. Kinase inhibitor binding may lock these interconversions in the inactive state. We have recently shown that FDA-approved kinase blockers along with lead molecules in (pre)clinical trials convert central kinase components of the mitogen-activated protein kinase (MAPK) pathway back into the inactive conformation [22, 33, 37].

First, we demonstrated that a collection of patient and activity-targeted kinase mutation of *BRAF* and *MEK1* converted KinCon reporters into the activated opened state of the full-length kinase. Second, we presented evidence that a selection of kinase inhibitors occupied the respective catalytic kinase cleft, blocked the phospho-transfer and led to the conversion of the activated KinCon reporter back into the closed conformation state [22, 33, 37]. As mentioned above, such drug or mutation driven reorganizations of enzyme structures may involve CRE or related auto-inhibitory regions to control their activity states [20]. For some other kinases, the concept of intramolecular pseudosubstrate motif interactions may contribute to kinase structure dynamics [11, 41]. Based on recent observations related to CRE/AIM predictions and novel Kin-Con reporter studies, we assume that a collection of kinases may engage trackable opened and closed full-length kinase configurations and thus activity states.

Previously, we demonstrated the generation of Kin-Con reporters for a collection of kinases [22, 30, 33, 37, 38]. However, for the construction of a KinCon biosensor, the following limitations need to be considered. Besides sizes of full-length kinases with more than 100kDa, the subcellular localization and transmembrane domains may complicate the design and implementation of a functional KinCon reporter. In addition, KinCon reporter which are localized to specific cell compartments such as the mitochondrial matrix, might be less accessible for luciferase substrates. Furthermore, addition of the PCA fragments to the kinase may interfere with cellular interactions and functions of the protein.

Overall, we assume that the systematic KinCon:drug profiling will open new possibilities for kinase-oriented drug discovery efforts: First, time- and dose-dependent exposures with lead molecules bear the chance to record activity-relevant full-length kinase conformations directly in the intact cells of choice. Thus, efficacies of single-agents or targeted combination therapies can be determined within a short timeframe to enable earlier therapeutic intervention [22, 33].

Second, these cell-based assays can be extended to implement cell-type specific PPIs, PTMs, and the particular kinase mutation into KinCon measurements. The exposing of cellular KinCon reporters to defined cell-type specific and molecular interactions should deliver novel insight into intramolecular kinase dynamics. Exemplarily, we recently described such allosteric effects of mutation-specific BRAFi on the molecular interactions of the mutated BRAF oncoprotein showing implications for the architecture of a tetrameric RAS:RAF complex [20, 22].

Third, another major advantage of the system is scalability that would allow high-throughput screens and the comparably quick adaption to novel patient mutations via site-directed mutagenesis. Thus, we believe that the KinCon biosensors have the potential to answer questions regarding precision medicine in terms of oncokinase mutation and pharmaceutical interference in a systematic manner. Fourth, we would like to pinpoint that for some kinases cellular read-outs for regulation-decoupled activities are still missing. In these lines, KinCon reporters may become suitable for analyzing cell-type specific features of pseudokinases. Besides constitutive phosphotransferase activities and alterations of abundance and localization, genetic alterations may also promote kinase inactivation. Indeed, it is a growing spectrum of kinase features which lead to enzyme dysfunctions contributing in different ways to disease etiology and progression [2, 29, 42–45]. In this context, KinCon reporters may become an asset to track and perturb the afore mentioned context-dependent cellular activity states. What is more, KinCon recordings of kinase conformation changes hold the promise to answer which type of lead molecule would work best to block the respective mutated kinase function.

Finally, KinCon reporter profiles may become relevant for laborious clinical studies by specifying the recruitment of patients with a specific mutation spectrum. Systematic KinCon biosensor measurements would anticipate which patients (displaying a particular oncokinase mutation) may respond with the highest efficacies to the treatments.

**Fig. 3:**
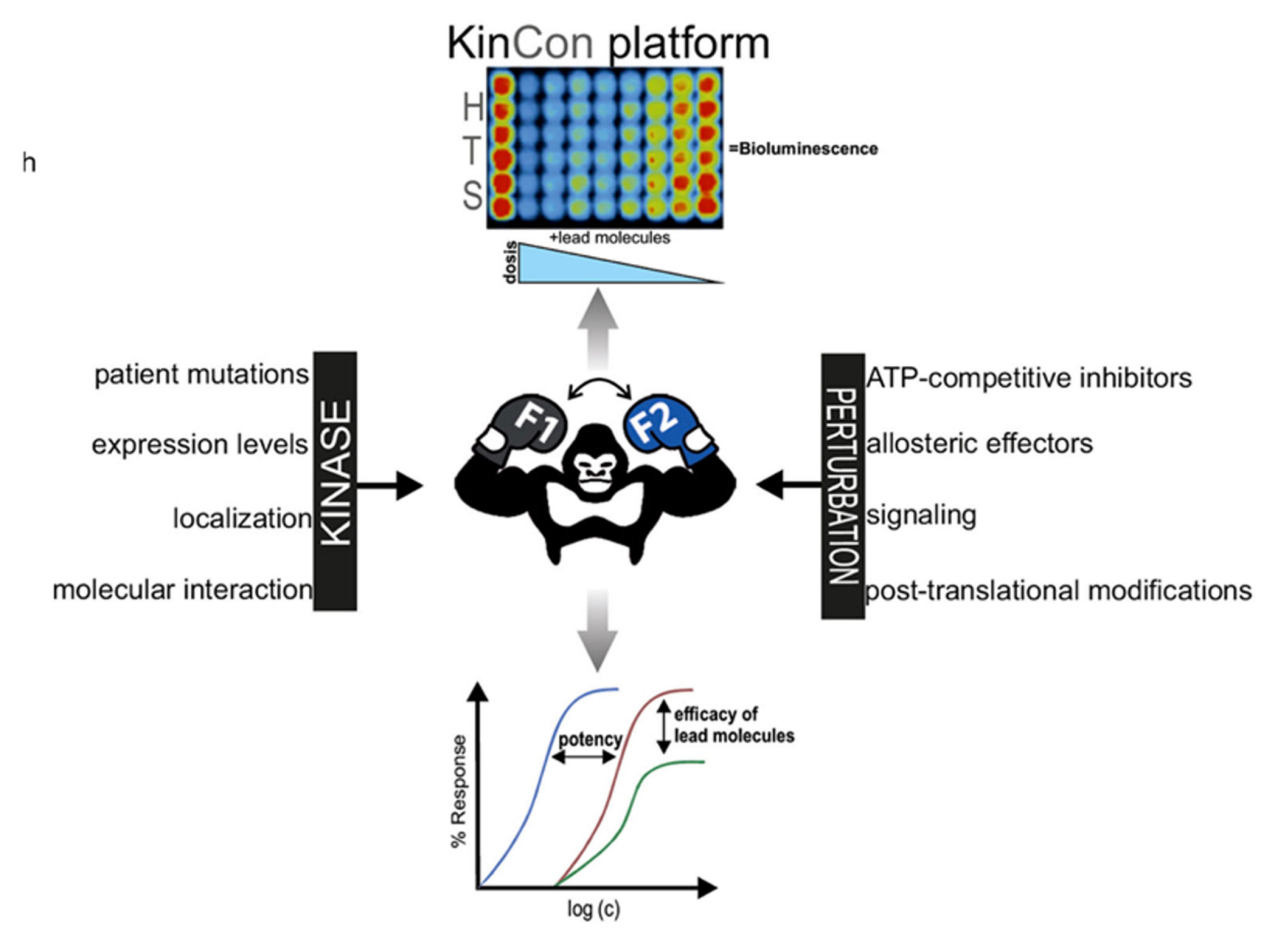
KinCon reporter dynamics. Cellular and bio-chemical features of kinases are indicated and perturbation measures (which are related to lead molecule interaction and/or signaling) are listed. Alterations of cellular KinCon dynamics are trackable via bioluminescence signals. Systematic quantifications of KinCon dynamics in high content format (*HTS* high through-put screening format) will ease the systematic determination of lead molecule efficacies and potencies (*c* concentration of the bioactive small molecule/lead molecule/drug), dependent of the genetic profile and diverse cellular features

Fig. 3 illustrates how cellular features of kinases may affect the respective KinCon states and thus kinase activities. Indicated perturbation strategies may reflect alterations of lead molecule efficacies and/or potencies in the selected cell culture setting and in high content format.
